# Prevalence and trends of markers of hepatitis B virus, hepatitis C virus and human Immunodeficiency virus in Argentine blood donors

**DOI:** 10.1186/1471-2334-14-218

**Published:** 2014-04-23

**Authors:** Diego M Flichman, Jorgelina L Blejer, Beatriz I Livellara, Viviana E Re, Sonia Bartoli, Juan A Bustos, Claudia P Ansola, Susana Hidalgo, Martin E Cerda, Alicia E Levin, Adriana Huenul, Victoria Riboldi, Elena MC Treviño, Horacio J Salamone, Felix A Nuñez, Robert J Fernández, Juan F Reybaud, Rodolfo H Campos

**Affiliations:** 1 Universidad de Buenos Aires, Cátedra de Virología, Buenos Aires, Argentina; 2 Fundación Hemocentro, Buenos Aires, Argentina; 3 Hospital Italiano Buenos Aires, Buenos Aires, Argentina; 4 Universidad Nacional de Córdoba, Córdoba, Argentina; 5 Centro regional de Hemoterapia Jujuy, San Salvador de Jujuy, Argentina; 6 Banco de sangre San Jorge, Ushuaia, Argentina; 7 Servicios de Hemoterapia de la provincia de Mendoza, Mendoza, Argentina; 8 Hospital Dr Enrique Vera Barros, La Rioja, Argentina; 9 Hospital Dr. Lucio Molas, Santa Rosa, Argentina; 10 Hospital Artémides Zatti, Viedma, Argentina; 11 Hospital Regional Rio Gallegos, Rio Gallegos, Argentina; 12 Banco de Sangre de la Universidad Nacional de Córdoba, Córdoba, Argentina; 13 Fundación Favaloro, Buenos Aires, Argentina; 14 Servicio de Hemoterapia del Hospital Italiano, Buenos Aires, Argentina; 15 Universidad de Buenos Aires, Cátedra de Virología, Junín 956 - 4 piso, ZIP code: 1113, CABA, Buenos Aires, Argentina

**Keywords:** Prevalence, Trend, Blood donors, HIV, HBV, HCV

## Abstract

**Background:**

Transfusion-transmitted infections are a major problem associated with blood transfusion. The aim of this study was to determine prevalence and trends of HBV, HCV and HIV in blood donors in Argentina.

**Methods:**

A retrospective study was carried out in blood donors of 27 transfusion centers covering the whole country over a period of eight years (2004-2011). Serologic screening assays for HBsAg, anti-HBc, anti-HCV, and anti-HIV were performed in all centers and nucleic acid amplification testing (NAT) was performed in 2 out of the 27 centers.

**Results:**

The 2,595,852 samples tested nationwide from 2004 to 2011 showed that the prevalence of HBsAg decreased from 0.336% to 0.198% (p < 0.0001), that of anti-HBc from 2.391% to 2.007% (p < 0.0001), that of anti-HCV from 0.721% to 0.460%, (p < 0.0001) and that of anti-HIV from 0.208% to 0.200 (p = 0.075). The prevalence of HBV, HCV and HIV was unevenly distributed among the different regions of the country. Two out of 74,838 screening- negative samples were positive in NAT assays (1 HIV-RNA and 1 HCV-RNA); moreover, HBV-DNA, HCV-RNA and HIV-RNA were detected in 60.29, 24.54 and 66.67% of screening-positive samples of the corresponding assays. As regards donors age, positive HBV-DNA and HCV-RNA donors were significantly older than healthy donors (46.6, 50.5 and 39.5 y respectively, p < 0.001).

**Conclusions:**

Argentina has a low prevalence of HBsAg, anti-HCV and anti-HIV in blood donors, with a decreasing trend for HBsAg, anti-HBc and anti-HCV but not for anti-HIV over the last 8 years. The uneven distribution of transfusion-transmitted infections prevalence among the different regions of the country highlights the need to implement regional awareness campaigns and prevention. The discrepancy between samples testing positive for screening assays and negative for NAT assays highlights the problem of blood donors who test repeatedly reactive in screening assays but are not confirmed as positive upon further testing. The uneven distribution of age between healthy donors and NAT-positive donors could be related to changes in risks of these pathogens in the general population and might be attributed to a longer exposure to transmission risk factors in elderly people.

## Background

Hepatitis B (HBV), hepatitis C (HCV) and human immunodeficiency (HIV) viruses, the three most important agents responsible for transfusion-transmitted infections (TTIs), are a global public health problem and still a large health care burden globally. It is currently estimated that about 350 million people worldwide are chronically infected with HBV, 170 million with HCV and 38 million HIV [1-5].

The evaluation of the prevalence and distribution of HBV, HCV and HIV is important for the planning of preventive measures and particularly, in the case of HBV, for the development of vaccination programs. The prevalence rates across the world are difficult to calculate given the asymptomatic and often latent nature of these diseases prior to clinical presentation [6,7]. The ideal condition to carry out a seroprevalence study is to sample the general population; however, this is not always feasible. Because of the low prevalence of these infections in the general population, their determination is cumbersome because it would imply large sample sizes. For this reason, although blood donors may not reflect the general population, most of the studies are performed in this group because results could be invaluable data to better understand the epidemiology of these diseases in the community [8-11].

In addition, the laboratory procedures and brands of reagents used in the different centers may differ in sensitivity and specificity; comparisons between them are not straightforward. In spite of these limitations, the information found is helpful, because in Argentina, there is a paucity of current epidemiological studies including an appropriate sampling of the general population [12].

In the last years, several studies have estimated the epidemiological burden of HBV, HCV and HIV infection in Argentina. Nonetheless, most of the data are outdated [13,14] or come from regional studies focused on small communities with an unrepresentative number of cases [15-18]. There are also studies limited to vulnerable populations or coinfected patients [19-22]. Therefore, there is a paucity of information about the current burden of HBV, HCV and HIV in Argentina.

In Argentina, altruistic repeat donors are the exception rather than the rule and most blood comes from new donors; feature that did not change significantly during the period in which the study was conducted [23]. This should be taken into consideration, since the prevalence of positive serologic tests for infectious diseases in these donors is higher than that among repeat volunteers, who are subjected to periodic screening [24,25].

A better understanding of the prevalence of TTIs can help medical communities and government agencies manage the disease burden and develop strategies in light of the emergence of several potent antiviral therapies. The present study should help to increase awareness of TTIs in the region.

In the present retrospective study, we evaluated the seroprevalence of HBV, HCV and HIV among blood donors in Argentina from 2004 to 2011. We also determined the trend of these infections and compared the prevalence in the different regions of the country.

## Methods

### Population

Blood donor records covering the period between 2004 and 2011 were analyzed. Data were collected from 27 transfusion centers throughout the country, 24 of which were public and 3 were private. In the course of the study, most (~90%) blood donors were volunteers or relatives or friends of the recipients, whereas the remaining ones were repeat volunteers, periodically subjected to screening.

Potential donors undergo a questionnaire and a physical examination performed by trained physicians. Those who are apparently healthy, are between 18-65 years old and weigh above 45 kg are qualified for donation.

The data were analyzed considering seven geographic regions: northwest (NW), northeast (NE), West, South, Center, Buenos Aires city (CABA) public and private centers, as well as the provinces that make up each region.

In addition, age and gender of 744 healthy donors randomly selected, from 2 centers where NAT assays are routinely performed, were recorded to compare the epidemiological features of NAT positive donors with those of non-reactive. The size of the healthy donor’s population was estimated by considering a confidence level of 95% and 1% of accuracy.

### Serologic assays

Serum samples were tested using third and fourth generation enzyme immune assays (EIAs) following the manufacturer’s instructions, for the presence of hepatitis B surface antigen (HBsAg), anti-Core antibody (anti-HBc), antibodies for hepatitis C and antibodies for HIV. The different centers used several methodologies, even in the same center, over the study period.

Trademarks used were, for HBsAg: PRISM HBsAg, ARCHITECT HBsAg, AxSYM HBsAg (Abbott Diagnostics, IL, USA), Murex HBsAg version 3 (Abbott Diagnostics, IL, USA) and Hepanostika HBsAg Ultra (Organon Teknika, Boxtel, The Netherlands). For anti-HBc: PRISM HBcore, ARCHITECT CORE (Abbott Diagnostics, IL, USA), Hepanostika anti-HBc Uniform (Organon Teknika, Boxtel, The Netherlands), Anti-HBc (Radim, Pomezia, Italy), Enzygnost Anti HBc (Dade Behring, IL, USA) and anti-HBc (Dia.Pro Diagnostic BioProbes, Milan, Italy). For anti-HCV: PRISM HCV, ARCHITECT anti-HCV (Abbott Diagnostics, IL, USA), HCV Version 3.0 (Ortho Clinical Diagnostics, NJ, USA), Bioelisa HCV 4.0 (Biokit, Lexington MA, USA), HCV Ab (DIA.PRO Diagnostic, Bioprobes srl, Milan, Italy) and Murex anti-HCV 4.0 (Murex Biotech Ltd., Dartford, United Kingdom) and for anti-HIV: PRISM HIV O Plus, ARCHITECT HIV Ag/Ab Combo, AxSYM HIV Ag-Ab Combination (Abbott Diagnostics, IL, USA), Murex HIV Ag-Ab Combination (Murex Biotech Ltd., Dartford, United Kingdom), HIV Ab&Ag 4th Gen (DIA.PRO Diagnostic, Bioprobes srl, Milan, Italy), VIDAS HIV Duo Ultra (Biomerieux, Marcy l’Etoile, France), GS HIV-1/HIV-2 PLUS O (Ortho Clinical Diagnostics, NJ, USA).

### Nucleic acid amplification testing (NAT) assays

The HBV, HCV and HIV nucleic acids were routinely tested in two of the 27 centers using Cobas Taqscreen MPX test (ROCHE) and Procleix Ultrio test (Chiron), following the manufacturer’s instructions. Positive samples were later analyzed with Procleix HIV-1, HCV and HBV Discriminatory assays or Cobas TaqScreen MPX Test, v2.0 to determine which viral nucleic acid or combination of nucleic acids was causing the reaction.

### Statistical analysis

Results were expressed as mean ± SD. Fisher’s two-tailed exact test and the corrected *X*2 test were used to compare qualitative data. ANOVA and non-parametric test (Mann-Whitney U and Kruskal-Wallis H) were used to compare quantitative variables. The data were entered into a computer and analyzed using Statistical Package for Social Sciences (SPSS, Chicago, IL, USA), version 16.0. Significance was set at a value of less than 0.05.

### Ethical aspects

This study was approved by the Ethics Committee of the School of Pharmacy and Biochemistry, Buenos Aires University (EXPTE. N° 732575). Interviews and blood sample collection were conducted after written consent forms had been signed. The study was performed in accordance with provisions of the Declaration of Helsinki and Good Clinical Practice guidelines.

## Results

### Population

During the 8-year study, the number of blood donors progressively increased from 352,771 in 2004 to 573,518 in 2011. The sampling in 2011 represented 1.38% of the country population, (41,660,417 inhabitants) and ranged between 0.73 and 2.10% in the different regions studied.

### HBV prevalence

In 2011, the nationwide prevalence of HBsAg was 0.198 ± 0.179% (0.090-0.725%), being unevenly distributed in the different regions of the country (Table 1).

**Table 1:** Prevalence of HBsAg, anti-HBc, anti-HCV and anti-HIV in 2011 by region and province

	**Donors**	**Prevalence (%)**			
		**HBsAg**	**Anti-HBc**	**Anti-HCV**	**Anti-HIV**
**NW**	**54,138**	**0.451**	**5.221**	**0.660**	**0.131**
Jujuy	9,740	0.671	7.378	0.385	0.147
Salta	16,229	0.725	10.136	0.678	0.285
Tucumán	14,746	0.206	1.929	0.857	0.053
Catamarca	2,874	0.319	2.252	0.457	0.012
La Rioja	3,832	0.272	2.293	1.015	0.330
Santiago del Estero	6,717	0.467	1.875	0.344	0.113
**NE**	**45,899**	**0.291**	**3.163**	**0.335**	**0.124**
Formosa	11,512	0.231	3.810	0.458	0.105
Chaco	12,041	0.125	1.473	0.297	0.115
Corrientes	12,821	0.176	1.153	0.295	0.089
Misiones	9,525	0.656	7.039	0.344	0.197
**South**	**23,344**	**0.223**	**1.570**	**0.446**	**0.247**
Neuquén	5,178	0.226	1.997	0.562	0.376
Rio Negro	5,503	0.250	0.717	0.276	0.130
Chubut	6,717	0.183	1.424	0.239	0.143
Santa Cruz	4,018	0.249	1.768	0.693	0.338
Tierra del Fuego	1,928	0.177	1.629	0.457	0.129
**West**	**25,600**	**0.280**	**1.702**	**0.434**	**0.131**
Mendoza	17,332	0.357	2.018	0.530	0.180
San Juan	4,971	0.128	1.192	0.117	0.022
San Luis	3,297	0.097	0.807	0.349	0.026
**Center**	**320,595**	**0.151**	**1.466**	**0.436**	**0.200**
Córdoba	57,360	0.155	1.512	0.456	0.221
La Pampa	4,250	0.246	0.791	0.499	0.193
Buenos Aires	209,281	0.112	1.313	0.462	0.219
Entre Ríos	16,483	0.132	1.259	0.195	0.063
Santa Fe	33,221	0.221	2.262	0.349	0.105
**CABA (public)**	**60,807**	**0.181**	**2.077**	**0.641**	**0.308**
**CABA (private)**	**43,135**	**0.090**	**1.085**	**0.287**	**0.104**
**TOTAL**	**530,383**	**0.198**	**2.007**	**0.460**	**0.190**

CABA: Buenos Aires city.

The highest prevalence rate was observed in the NW region (0.451%); particularly, among the provinces belonging to this region, Salta (0.725%) and Jujuy (0.671%) showed prevalence rates higher than the other provinces of this region (Tucuman 0.206%, Catamarca 0.319%, La Rioja 0.272% and Santiago del Estero 0.467%).

The other regions showed similar prevalence rates, ranging from 0.090 to 0.291%. However, although the rate in the NE region was within the average (0.291%), the prevalence in the province of Misiones (0.656%), belonging to this region, was significantly higher, only comparable to that in Salta and Jujuy. Finally, the prevalence of HBsAg was significantly different between CABA public centers and CABA private centers (0.181% and 0.090% respectively, p < 0.001).

The anti-HBc prevalence broadly followed the HBsAg rates, being the national prevalence 2.007 ± 2.308% (range 0.717-10.136%). The provinces with higher HBsAg rates (Salta, Jujuy and Misiones) showed high prevalence rates of anti-HBc (> 7.0%). The average ratio of anti-HBc/HBsAg was 9.87 ± 2.29, ranging from 6.074 to 12.00 in the different regions.

**Figure 1:**
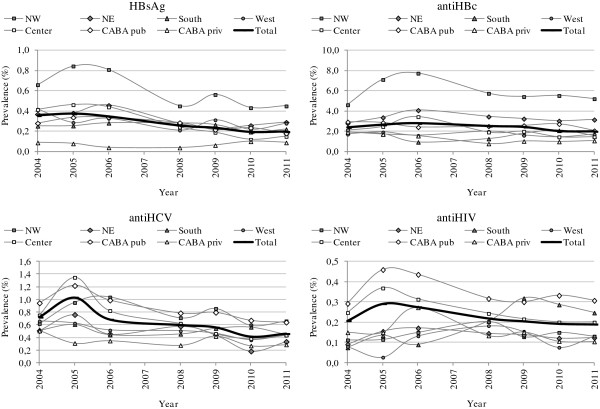
**Trend in prevalence of HBsAg, anti-HBc, anti-HCV and anti-HIV in blood donors from 2004 to 2011.** CABA: Buenos Aires city.

Along the study period, we found a slight and significant decrease at nationwide level for HBsAg prevalence from 0.356% in 2004 to 0.198% in 2011 (p < 0.0001) and for anti-HBc prevalence from 2.391% in 2004 to 2.007% in 2011 (p < 0.0001) (Table 2). This pattern was observed in four out of the seven regions studied (NW, West, Center and CABA public centers) whereas it remained steady in the other three regions (NE, South and CABA private centers) (Figure 1).

**Table 2:** Comparison of prevalence rates between 2004 and 2011 in the different regions for each serological marker

	**HBsAg**			**Anti-HBc**			**Anti-HCV**			**Anti-HIV**		
**Region**	**2004**	**2011**	**p**	**2004**	**2011**	**p**	**2004**	**2011**	**p**	**2004**	**2011**	**p**
NW	0.660	0.451	**0.001**	4.623	5.221	**0.004**	0.618	0.660	0.572	0.112	0.131	0.578
NE	0.371	0.291	0.054	2.809	3.163	**0.004**	0.509	0.335	**<0.001**	0.091	0.124	0.164
South	0.254	0.223	0.560	1.895	1.570	**0.025**	0.513	0.446	0.351	0.075	0.247	**<0.001**
West	0.419	0.280	**0.009**	1.728	1.702	0.823	0.659	0.434	**<0.001**	0.080	0.131	0.068
Center	0.416	0.151	**<0.001**	2.110	1.466	**<0.001**	0.753	0.436	**<0.001**	0.247	0.200	**<0.001**
CABA public	0.282	0.181	**<0.001**	2.946	2.077	**<0.001**	0.945	0.641	**<0.001**	0.292	0.308	0.617
CABA private	0.090	0.090	1.000	1.945	1.085	**<0.001**	0.507	0.287	**<0.001**	0.150	0.104	0.070
TOTAL	0.336	0.198	**<0.001**	2.391	2.007	**<0.001**	0.721	0.460	**<0.001**	0.208	0.190	0.066

### HCV prevalence

In 2011, the nationwide prevalence of anti-HCV was 0.460 ± 0.207% (0.117-1.015%), being significantly higher in the NW region (0.660%) and CABA public centers (0.641%) than in the other regions (Table 1). In particular, among the provinces belonging to the NW region, Salta (0.678%), Tucumán (0.857%) and La Rioja (1.015%) showed significantly higher prevalence rates than the other provinces of this region. The other regions showed similar prevalence rates ranging from 0.287 to 0.446%. Similarly to that observed for HBsAg, the prevalence of anti-HCV in the CABA public centers was significantly different from that in the CABA private centers, being 0.641% and 0.287% respectively (p < 0.001).

In the longitudinal study between 2004 and 2011, the nationwide level of anti-HCV showed a significant decrease from 0.721% in 2004 to 0.460% in 2011 (p < 0.0001) (Table 2). This pattern was observed in five out of seven regions studied (NE, West, Center, CABA public and CABA private); whereas it remained unchanged in the other two regions (NW and South) (Figure 1).

### HIV prevalence

In 2011, the nationwide prevalence of anti-HIV was 0.190 ± 0.103% (0.012-0.376%). Overall, the prevalence by regions ranged from 0.104 to 0.247% except in CABA public centers (0.308%), where it was significantly higher than in the other regions (Table 1). In some provinces as, La Rioja (0.330%), Neuquén (0.376%) and Santa Cruz (0.338%) the prevalence was above the average.

The prevalence of anti-HIV in the public centers of CABA was significantly different from that in the private centers of CABA, being 0.308% and 0.104% respectively (p < 0.001).

Along the study period, the nationwide prevalence of anti-HIV between 2004 and 2011 was unchanged (0.208 to 0.190%; p = 0.075) (Table 2). It only showed a significant decrease in the Center (0.247 to 0.200%; p < 0.001) and a significant increase in the South (0.075 to 0.247%; p < 0.001) (Figure 1).

### Nucleic acid amplification testing (NAT)

During 2010 and 2011, 75,200 donors were routinely tested by NAT assays in two private centers CABA; 74,838 of these were negative in serologic assays and 362 were positive in serology assays for any of transfusion-transmissible agents. Two out of 74,838 screening- negative samples were positive in NAT assays: one for HCV-RNA and the other for HIV-RNA.

**Figure 2:**
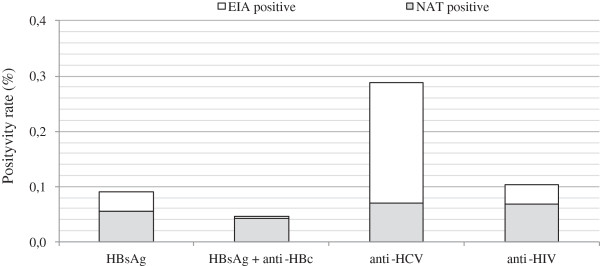
**Positivity rates in screening test and NAT assays.** In the years 2010 and 2011 75,200 samples were analyzed by screening test and NAT assays in two CABA transfusion centers. White bars: screening-positive test, gray bars: NAT-positive test.

On the other hand, when screening -positive samples were analyzed, HBV-DNA was detected in 41 out of 68 (60.29%) HBsAg-positive samples (Figure 2). The rate of HBV-DNA was significantly higher in those samples also reactive for anti-HBc (94.12%, 32 of 34) than in samples with HBsAg alone (24.47%, 9 of 34) (p = 8.8e-^9^). While, HCV-ARN was detected in 53 out of 216 anti-HCv positive samples (24.54%) and HIV-RNA in 52 out of 78 anti-HIV positive samples (66.67%).

**Figure 3:**
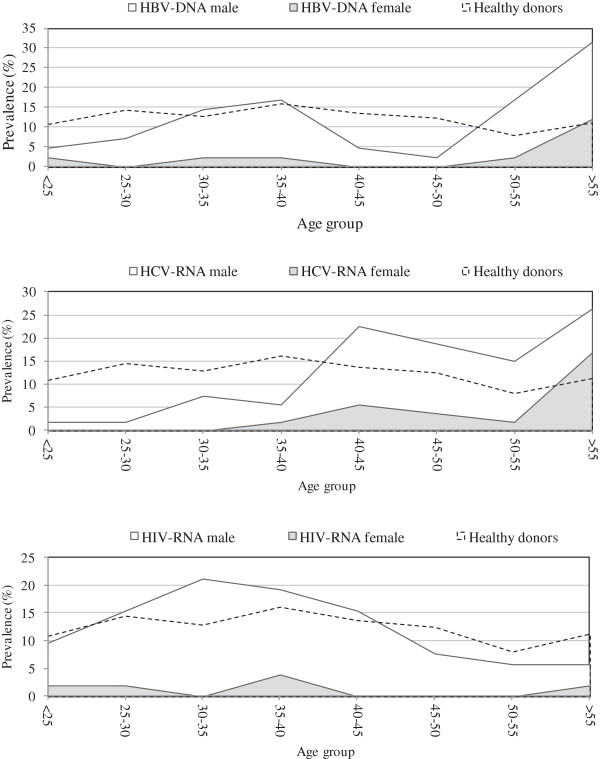
**Frequency of positive HBV-DNA, HCV-RNA and HIV-RNA by age.** Gray areas: NAT-positive female; white areas: NAT-positive male; dashed line: healthy donors.

Finally, when comparing healthy donors and NAT-positive samples by age, HBV-DNA positive donors were significantly older than healthy donors (46.6 ± 14.8 vs 39.5 ± 11.7, p = 0.001) (Table 3). Most of the positive samples (48.8%) come from donors over 50 years of age, while only 19.3% of healthy donors were in this age range (Figure 3). As regards HCV-RNA positive donors, age distribution was also significantly higher (50.5 ± 14.3, p < 0.0001) than that of healthy donors (Table 3); particularly, there was a clear cut of positivity frequency between donors younger than 40 years (17.0%) and those over 40 years (83.0%) (Figure 3). Finally, the age distribution of HIV-RNA-positive donors was not significantly different from that of healthy donors (37.1 ± 9.7, p = 0.168). However, the highest incidence was observed in the 30-40 age range (40.4%) (Figure 3).

**Table 3:** Comparison of age and gender between healthy donors and NAT positive donors

	**Healthy donors**	**HBV-DNA**	**Anti-HBc**	**HCV-RNA**	**HIV-RNA**
n	744	41	254	53	52
Ratio m:f	1.9	3.6	3.4	2.1	9.4*
Mean age (y)	39.5	46.6*	47.0*	50.5**	37.1
SD (y)	11.7	14.8	14.3	14.4	9.7

*p < 0.001, **p < 0.0001.

## Discussion

In this study, we characterized the prevalence and trend of HBsAg, anti-HBc, anti-HCV and anti-HIV in blood donors in Argentina between 2004 and 2011.

Although most studies on the seroprevalence of TTIs come from blood centers, these data are biased. Data may be either underestimated -because blood donor candidates are preselected based on a questionnaire and a physical exam and blood is drawn only from those who appear at low risk of having blood-borne pathogens- or overestimated -given that, as in most studies performed in blood banks, positive screening tests are not confirmed and have a variable percentage of false positives.

Nevertheless, by comparing several studies, Petrovic showed that the ratio of prevalence between general population and blood donors ranges from 1.17 to 1.70 for HBsAg and from 1.25 to 3.00 for anti-HCV [26]. Moreover, in a study conducted by Karaosmanoglu, a ratio of 1.89 was observed when comparing the prevalence of HBsAg among blood donors and healthy people who required a premarital screening [27]; whereas in a study performed by Baha, where HBV and HCV prevalence in general population and blood donors was compared, a ratio of 2.55 and 1.89 for HBV and HCV, was observed [28]. Therefore, although the seroprevalence determined from blood donors may not reflect that of the general population, it provides a better understanding of the epidemiology of these diseases in the community [29].

Overall, our results show that the current prevalence of HBsAg, anti-HCV and anti-HIV in blood donors in Argentina is relatively low. The HBsAg and anti-HCV prevalence was unevenly distributed across the country, both between and within regions. This suggests the need to implement regional awareness campaigns and prevention according to the severity of the problem, to reduce the risk of transmission of infection. The relationship between anti-HBc/HBsAg indicates a chronicity rate below 10%, suggesting that the transmission of HBV in Argentina occurs mostly during adulthood.

The common finding that the prevalence of serologic markers of infection was higher in donors from the public blood banks suggests, as previously stated by Schmuñis [18], a difference in the socioeconomic status of the donors, which is usually associated with an increased condition of vulnerability.

Although blood bank data may underestimate the true prevalence, it is likely that the trend in the donors provides a conservative estimate of the trend in the general population [30]. The average rates of HBsAg and anti-HCV in most of the regions and nationwide decreased significantly over time between 2004 and 2011. These results are in agreement with the trend observed in our country by Schmuñis in the 1990s [18] and may be the consequence of increased public consciousness and effective use of preventive measures, particularly in HBV because of the implementation of vaccination plans. However, it could also be partly due to the improvement in the specificity of diagnostic tests during this period. On the other hand, anti-HIV prevalence did not change significantly over the study period.

Although HBV, HCV and HIV share the transmission routes, and even though the sexual transmission of HCV is not very efficient, the different trends observed in the last years between HBV and HCV compared with HIV prevalence could be due to prevention and awareness measures mainly associated with those factors that are more preponderant for HBV and HCV infections, such as IVDU or socioeconomic status.

NAT assays are usually done to reduce the window period and consequently reduce the risk of TTIs; nonetheless, they are also useful to assess the specificity of serologic screening tests. An increase in screening test sensitivity of TTIs is highly desirable to ensure recipient safety. However, in populations with a low prevalence rate, the positive predictive value is relatively poor [31-33].

Viral nucleic acids were not detectable in a significant number of reactive samples in screening assays (Figure 2). This suggests that, regardless of the sensitivity of NAT assays, most of these samples were false positive. Actually, a major problem facing blood banks today is the loss of donors who test repeatedly reactive in screening enzyme immunoassays but are not confirmed as positive upon further testing [34].

As regards HBV, many authors have observed low specificity for total anti-HBc tests when using enzyme immunoassays [32,35,36]. However, the use of this immunoassay as additional marker significantly increased the positive predictive value of the identification of truly HBV-infected individuals. In the case of HCV, it is estimated that between 15 and 25% of infections are self-limited, a percentage that varies depending on the HCV genotype. Therefore, the false positive rate, although high, is probably lower than 75.46%. Considering 15 to 25% of self-limited infections, the false positive rate would range from 67.28 to 71.13%. Similar results have been observed in previous studies by comparing EIA screening assays with confirmatory assays [37-39]. Finally, the test for HIV showed the highest specificity when compared with NAT assays. These results are consistent with those obtained in previous studies [32,33,40].

Overall, our results suggest that the true prevalence in blood donors, particularly for HBV and HCV, is significantly lower than that determined by considering the screening tests.

The uneven distribution of age between healthy donors and NAT-positive donors could be related to changes in risks of these pathogens in the general population. Teenage children and young adults in the 1960s and 1970s (who were over 40 years old between 2010 and 2012) might have received transfusions or medical procedures with non-disposable needles and thus might have become infected with HCV. The increase in HIV prevalence among donors between 30 and 40 years of age since 2000 could reflect the change in perception among the public about HIV threat and could have therefore led to a more relaxed control of HIV transmission. For example, some people might have mistakenly believed that available treatment for HIV infection is effective and thus might have engaged in more risky behavior, which could have resulted in the recent increase in new infections among men who have sex with men in certain areas [41,42]. These results are consistent with those obtained in previous studies [25,28,43].

From our results, it is evident that the status of the blood supply in Argentina improved steadily from 2004 through 2011 and follows the trend described by Schmuñis from 1995 to 1997 [18].

Despite the limitations of the data discussed above, it is clear from the analysis that there is a need to establish a continued monitoring system to detect pitfalls that need remedy and to gain support to strengthen blood banking activities and assess the level and quality of screening for infectious diseases in the blood supply.

## Conclusions

The present study provides an updated description of the prevalence and trends of HBV, HCV and HIV in blood banks in Argentina. The analysis by region and province; allowed to understand the different prevalence and trend scenarios of these transfusion-transmitted agents throughout the country; highlighting the need to implement regional awareness campaigns and prevention according to the severity of the problem. The longitudinal analysis of the data allowed to determine the trend of transfusion-transmitted infections; emphasizing an overall decrease in the prevalence of HBV and HCV infections in the last years. Moreover, the comparative analysis of EIA and NAT assays, conducted in two centers, allowed assessing, for the first time in our country, -the impact of implementing this methodology in the routine screening, -the specificity of screening assays and -the usefulness of including the antiHBc test in the screening.

## Abbreviations

TTIs: Transfusion-transmitted infections; HBV: Hepatitis B virus; HCV: Hepatitis C virus; HIV: Human immunodeficiency virus HBsAg, Hepatitis B surface antigen; antiHBc: Anti-Core antibody; Anti-HCV: Anti-HCV antibody; EIA: Enzyme immune assays; NAT: Nucleic acid test; NW: Northwest; NE: Northeast; CABA: Buenos Aires city.

## Competing interests

The authors declare that they have no competing interests.

## Authors’ contributions

DF designed the study, collection the data, performed the statistical analysis and drafted the manuscript; JB, BL, VR, SB, JB, CA, SH, EMC, AL, AH, VR, ET, JHS, FN, JRF, and JR contributed to the acquisition of data and provided the source databases, JB participated in the data analysis and revised critically the manuscript, RHC revised the analysis plan and made an important intellectual contribution in the content. All authors read and approved the final version of this manuscript.

## Pre-publication history

The pre-publication history for this paper can be accessed here:

http://www.biomedcentral.com/1471-2334/14/218/prepub
